# Association of the *ACTN3* rs1815739 Polymorphism with Physical Performance and Injury Incidence in Professional Women Football Players

**DOI:** 10.3390/genes13091635

**Published:** 2022-09-12

**Authors:** Juan Del Coso, Gil Rodas, Miguel Ángel Buil, Javier Sánchez-Sánchez, Pedro López, Joaquín González-Ródenas, Pablo Gasulla-Anglés, Álvaro López-Samanes, Sergio Hernández-Sánchez, Ane Iztueta, Víctor Moreno-Pérez

**Affiliations:** 1 Centre for Sport Studies, Rey Juan Carlos University, 28943 Fuenlabrada, Spain; 2 Medical Department & Barça Innovation Hub, Fútbol Club Barcelona, 08028 Barcelona, Spain; 3 Department of Sports Medicine, Levante Unión Deportiva, 46360 Valencia, Spain; 4 Department of Sports Medicine, IVRE—Institut Valencià de Recuperació Esportiva, 46010 Valencia, Spain; 5 Faculty of Sport Sciences, Universidad Europea de Madrid, 28670 Madrid, Spain; 6 Medical Department, Valencia Club de Fútbol, 46980 Paterna, Spain; 7 Medical Department, Villarreal Club de Fútbol, 12540 Vilarreal, Spain; 8 Faculty of Health Sciences, Universidad Francisco de Vitoria, 28223 Pozuelo de Alarcón, Spain; 9 Center for Translational Research in Physiotherapy, Department of Pathology and Surgery, Miguel Hernandez University of Elche, 03202 Elche, Spain; 10 Health and Performance Unit, Real Sociedad de Fútbol Sociedad Anónima Deportiva, 20160 Donostia, Spain

**Keywords:** athletic performance, exercise-related injury, single nucleotide polymorphism, α-actinin-3 deficiency

## Abstract

The p.R577X polymorphism (rs1815739) in the *ACTN3* gene causes individuals with the XX genotype to be deficient in functional α-actinin-3. Previous investigations have found that XX athletes are more prone to suffer non-contact muscle injuries, in comparison with RR and RX athletes who produce a functional α-actinin-3 in their fast-twitch fibers. This investigation aimed to determine the influence of the *ACTN3* R577X polymorphism on physical performance and injury incidence of players competing in the women’s Spanish first division of football (soccer). Using a cross-sectional experiment, football-specific performance and epidemiology of non-contact football-related injuries were recorded in a group of 191 professional football players. *ACTN3* R577X genotype was obtained for each player using genomic DNA samples obtained through buccal swabs. A battery of physical tests, including a countermovement jump, a 20 m sprint test, the sit-and-reach test and ankle dorsiflexion, were performed during the preseason. Injury incidence and characteristics of non-contact injuries were obtained according to the International Olympic Committee (IOC) statement for one season. From the study sample, 28.3% of players had the RR genotype, 52.9% had the RX genotype, and 18.8% had the XX genotype. Differences among genotypes were identified with one-way analysis of variance (numerical variables) or chi-square tests (categorical variables). Jump height (*p* = 0.087), sprint time (*p* = 0.210), sit-and-reach distance (*p* = 0.361), and dorsiflexion in the right (*p* = 0.550) and left ankle (*p* = 0.992) were similar in RR, RX, and XX football players. A total of 356 non-contact injuries were recorded in 144 football players while the remaining 47 did not sustain any non-contact injuries during the season. Injury incidence was 10.4 ± 8.6, 8.2 ± 5.7, and 8.9 ± 5.3 injuries per/1000 h of football exposure, without differences among genotypes (*p* = 0.222). Injury rates during training (from 3.6 ± 3.7 to 4.8 ± 2.1 injuries per/1000 h of training exposure, *p* = 0.100) and match (from 47.8 ± 9.5 to 54.1 ± 6.3 injuries per/1000 h of match exposure, *p* = 0.209) were also similar in RR, RX, and XX football players. The *ACTN3* genotype did not affect the mode of onset, the time needed to return to play, the type of injury, or the distribution of body locations of the injuries. In summary, women football players with different genotypes of the p.R577X *ACTN3* polymorphism had similar values of football-specific performance and injury incidence. From a practical perspective, the *ACTN3* genotyping may not be useful to predict performance or injury incidence in professional women football players.

## 1. Introduction

α-actinin-3, encoded by the *ACTN3* gene, is a structural protein of the muscle fiber with a key role at the Z-disc, as it anchors actin filaments to maintain the myofibrillar array and regulate muscle length and tension during muscle contraction [1,2]. α-Actinin-3 is only expressed in fast muscle fibers, which suggests that the role of this protein is specific for fast muscle contractions or those performed with high levels of strength [3]. A common genetic polymorphism in the *ACTN3* gene (rs1815739, c.1858C>T), habitually known as the R577X polymorphism, leads to the replacement of an arginine (R) with a premature stop codon (X) at amino acid 577. Homozygous individuals for the X allele in the R577X polymorphism (XX genotype) produce a non-functional α-actinin-3, and they are habitually considered α-actinin-3-deficient [4]. In contrast, homozygous individuals for the R allele (RR genotype) or heterozygote individuals (RX genotype) express functional α-actinin-3, although it has been suggested that the expression of α-actinin-3 is higher in RR than in RX individuals [5]. The lack of α-actinin-3 is not linked with any disease as the α-actinin-3 deficiency is compensated by a higher expression of α-actinin-2, an α-actinin isoform ubiquitously expressed in all muscle fiber types. However, it has been demonstrated that *ACTN3* XX individuals possess several negative phenotypes, such as lower muscle strength [6] and muscle volume [7], impaired capacity to tolerate muscle strain [8] and decreased bone mineral density [9].

In sport, the *ACTN3* R577X polymorphism is one of the most investigated genetic variations, as several investigations have found that XX athletes are underrepresented in elite power-oriented athletes [2,10,11]. Although the exact phenotype(s) that cause(s) the underrepresentation of XX in power sport are/is not fully identified, the lower physical performance in sprint activities [12], the higher values of exercise-induced muscle damage [13] and a higher tendency for muscle injuries in XX, in comparison to RR athletes [14] may partially contribute. Among the power-oriented sports, football (soccer) is an ideal scenario to investigate the *ACTN3* R577X polymorphism as football is a “power/’explosive” sport and is characterized by the repetition of high-intensity actions, such as sudden accelerations, decelerations, sprints, changes of direction, jumping, and landings [15]. A current meta-analysis including 17 studies about the influence of the R577X polymorphism on the athlete status in football concluded that the RR genotype was overrepresented, while the presence of XX players is lower than in control non-athlete populations [16]. Additionally, in a population of elite Chinese female football players, it has been found that no player had the *ACTN3* XX genotype [17]. These outcomes suggest that the XX genotype may constitute a potential limitation to becoming an elite professional football player. However, these investigations only measured the proportions of each R577X genotype in samples of professional football players, while no football-specific performance phenotypes were measured.

The influence of the *ACTN3* R577X polymorphism on the injury rate in professional football players is another topic of investigation. Several studies have found that players with the XX genotype have certain susceptibility to developing non-contact musculoskeletal injury [18,19] and need more recovery time to return to play after this type of injury [20]. Probably, the lower values of quadriceps and hamstrings isokinetic strength in XX than RR players constitute a disadvantage to prevent muscle injuries in football [21], as most of the non-contact muscle injuries in football are located at the thigh, with a particularly high incidence in hamstring muscle [22]. However, these findings have been found in samples of male professional football players, while the influence of the *ACTN3* XX genotype on the probability of non-contact muscle injury—or any other type of injury— has not been tested in professional women football players.

The purpose of this investigation was to determine the influence of the *ACTN3* R577X polymorphism on physical performance variables and the injury incidence of professional players competing in the women’s Spanish first division of football (soccer). We hypothesized that XX players would have lower jump and sprint performance and a higher injury rate of muscle-type injuries than RR players.

## 2. Materials and Methods

### 2.1. Participants

One hundred and ninety-three professional football players volunteered to participate in the study. Participants played for any of the nine teams that competed in the women’s Spanish first division of football (Primera Iberdrola) in the 2020–2021 season that agreed to participate in the investigation. The study sample included football players of the team that won the championship and players from five teams ranked among the top ten positions of the championship. Players trained for an average of 4.7 ± 1.7 h/week during the season and performed ~1 competitive match per week, for a total of 30 official matches during the whole season. From the initial sample, two participants were excluded because their *ACTN3* genotype was not clearly identified in the genotyping analysis. Age, anthropometric characteristics, field position, and football exposure during the 2020–2021 season of the final sample of 191 professional football players are depicted in Table 1. All participants included in this investigation were Caucasian. The study protocol conformed to the Declaration of Helsinki for Human Research of 1974 (last modified in 2013) and was approved by the University Ethics Committee of Miguel Hernández University. Written informed consent was obtained from all participants before the onset of the experiment. Participants’ rights and confidentiality were protected during the whole experiment, and the genetic information was used only for the purposes included in this investigation.

### 2.2. Experimental Design

This investigation is a cross-sectional study to determine the effect, if any, of the *ACTN3* R577X genotype (RR vs. RX vs. XX) on football-specific physical performance and injury incidence of sport-related injuries suffered by professional football players during one season. For this investigation, a battery of physical performance tests was performed in the pre-season and the injuries resulting from their training routines or competitions were recorded during the whole 2020–2021 season. The questionnaire used to collect the injuries was based on the consensus statement on injury definitions and data collection in epidemiological studies of the International Olympic Committee [23].

### 2.3. Sample Collection and Genotyping

The DNA samples were collected through buccal smears between January and June 2020. A researcher went to the training facility of each football team to explain the aim of the investigation, its benefits, and risks, to obtain the written informed consent of participants willing to take part in the study, and to achieve two buccal swab samples per player for DNA analysis. During this process, the medical staff of the teams assisted the researcher. After collection, the samples were refrigerated at 4 °C and shipped to the laboratory. Upon arrival at the laboratory, the extraction of genomic DNA from the samples was carried out by automatic extraction in QIACube equipment (QIAGEN, Venlo, The Netherlands) to obtain a solution with a DNA concentration of 25–40 ng/mL. The solution was frozen at −20 °C until genotyped, which was done once all the samples had been received. During the genotyping process, the p.R577X polymorphism (rs1815739; c.1858C>T) in the *ACTN3* gene was genotyped using single nucleotide primer extension (SNPE). For this process, the SNaPshot Multiplex Kit (Thermo Fisher Scientific, Waltham, MA, USA) was used with capillary electrophoresis fragment analysis in ABI3500 equipment (Applied Biosystems, Foster City, CA, USA). Genotyping of *ACTN3* rs1815739 polymorphism was conducted using a TaqMan SNP Genotyping Assay (Applied Biosystems, CA, USA) and the reaction was performed in an Applied Biosystems 7500 Fast Real-Time PCR System (Applied Biosystems). All analysis that did not offer a clear genotype assignment were repeated. If the assignment of a genotype was still unsuccessful, the participant was removed for the analysis. The PCR was performed according to the previously published method [24]. Positive controls for all genotypes were obtained from the Mexican branch of the CANDELA Consortium. Fifty samples randomly selected samples were genotyped by duplicate, and we confirmed that the genotyping results were perfectly agreed between duplicates.

### 2.4. Physical Performance Testing

During the pre-season of the 2020–2021 season, football players performed a battery of performance tests. The performance tests were administered by the strength and conditioning staff of each team and were performed following the same procedures, including materials, order, and recovery between tests (>5 min). All participants were familiarized with the tests as they were part of the team’s physical assessments to assess players’ general physical conditioning after the transition period. All performance tests were performed within the same day and 24 h after a training day of low intensity. On the day of testing, anthropometric data were collected, and players completed a standardized warm-up consisting of 8 min of light-to-moderate runs, lower-limb dynamic stretching and sub-maximal attempts of sprinting and jumping tests. After this, participants performed the following tests.

#### 2.4.1. Ankle Dorsiflexion

The ankle dorsiflexion range of movement (ROM) was assessed on both ankles using the Leg Motion system test (LegMotion, Check your Motion, Albacete, Spain) and the procedures of Calatayud et al. [25]. For this measurement, players were in a standing position on the Leg Motion system with the foot to be measured on the measurement scale. The contralateral foot was placed outside the platform with the toes on its edge. Each player performed the test with their hands on their hips, with the assigned foot on the middle of the longitudinal line and just behind the transversal line of the platform. While maintaining this position, the players were instructed to flex the knee forward placing it in contact with a metal stick. The maximal distance obtained with the knee flexion was recorded while the player maintained the heel in contact with the platform. Three trials were allowed for each ankle with 30 s of passive recovery between trials. The best score for each ankle among these trials was selected for subsequent analysis.

#### 2.4.2. Sit-and-Reach Test

The sit-and-reach test was performed using the procedures outlined by the American College of Sports Medicine in their manual for guidelines for exercise testing and prescription [26]. A standard sit-and-reach box was used to position the players for the test, and a sliding ruler centered on the top of the box was used to obtain the scores. Each player sat on the floor with shoes off, with their legs together, knees fully extended, and soles of the feet placed against the end of the box. Placing one hand on top of the other, with palms down, participants then reached forward sliding their hands along the measuring scale. Two trials were performed, and the best result was used for statistical analysis.

#### 2.4.3. Countermovement Jump

The height reached during a bilateral countermovement jump (CMJ) was measured using a contact-time platform (Tapeswitch Signal Mat, Tapeswitch Corporation America, Farmingdale, NY, USA), following the procedures described by Lara et al. [27]. During the CMJ, participants were instructed to keep their hands on their hips and to jump as high as possible. Each player performed two maximal CMJs interspersed with 60 s of passive recovery. The best height for each player was recorded and used for statistical analysis.

#### 2.4.4. Sprint Time

The time during a maximal velocity 30 m sprint in a straight line was measured using photocells (Witty System, Microgate, Bolzano, Italy), following the protocol described by Vescovi for a 35 m sprint [28]. Each sprint was initiated 50 cm behind the photocell. Players started the sprint test in a standing position, with their preferred foot behind the starting line, followed by accelerating forward at maximal effort until they have passed the last photocell gate placed at 30 m. Each player performed two maximal 30 m sprints, with at least 2 min of passive recovery in between the two trials. The test was performed on the football pitch and participants wore their football cleats. The fastest time was used for statistical analysis.

### 2.5. Exposure Times

During the season, the strength and conditioning staff of each team meticulously registered players’ exposure in training and matches as part of their routines to estimate players’ load across the season. The warm-up of training activities and matches, the time employed for strength training activities, and the time during friendly matches were considered training exposure. Match exposure was computed as the sum of the time that each player was on the pitch during official matches of the national league and during international competitions. The strength and conditioning staff of each team sent a report with individual training and match exposure times to the researchers at the end of the season.

### 2.6. Injury Data Collection

In each team, all non-contact injuries were recorded by the medical services of the team during the 2020–2021 season (from 1 September 2020 to 30 June 2021). For the recording of injuries, the medical staff used an ad hoc questionnaire created by the research group of this study, which was filled once a football-related injury occurred. Injuries caused due to collision with another player or with an object (either direct or indirect) were excluded from the investigation as they are potentially unaffected by the player’s genotype. Data on injury epidemiology were obtained prospectively, and all injuries were diagnosed, classified, and recorded by the medical staff of the football clubs using the classification system developed by the medical commission of the International Olympic Committee (IOC) [23]. In each injury, the type of injury, the type of exposure, where it was sustained (training or match), the existence of recurrence, the mode of onset, and the body location were meticulously reported. Once the player returned to play with the group, the injury file was closed. We used the definition of the IOC consensus statement to classify injury severity by using data from the day of the injury and the day of their return to playing. Afterwards, injuries were grouped as slight (1–3 days), mild (4–7 days), moderate (8–28 days), or severe (>28 days). The medical staff of each team sent a report at the end of the season with the number and characteristics of the injuries sustained by each player. Injury incidence, in numbers of injuries per 1000 h of football exposure, was individually calculated for each football player using the number of non-contact injuries and football exposure time. Injury incidence during matches and training were also calculated separately using data on the number of injuries and exposure times in each scenario.

### 2.7. Statistical Analysis

Data on players’ genotype and performance and injury variables were introduced into an ad hoc database for analysis with the SPSS software package v.27.0 for Windows (IBM Corp., New York, NY, USA). The normality of each variable was initially tested with the Kolmogorov–Smirnov test, and parametric/non-parametric statistics were performed for normally/non-normally distributed variables, respectively. A chi-square (χ2) test was used to verify that the genotype frequencies were in Hardy–Weinberg equilibrium (HWE). A χ2 test was also used to verify if the genotype frequency in our cohort of football players complied with Hardy–Weinberg, with respect to the 1000 genome database of ethnically matched controls [29]. For the continuous variables, genotype comparisons (RR vs. RX vs. XX) were performed using a one-way analysis of variance (ANOVA; followed by Tukey’s posthoc comparisons) or the Kruskal–Wallis test. For the variables presented as frequency, the differences in distribution among genotypes were identified with crosstabs and χ2 tests, including adjusted standardized residuals. When comparing the dominant (RR vs. X-allele carriers) and recessive (XX vs. R-allele carriers) models, the differences in the distribution of injury characteristics were tested with χ2 tests and the differences in injury incidence were calculated with unpaired *t*-tests. The quantitative variables are presented as mean and SD (standard deviation) and the qualitative variables are presented by number and frequency.

## 3. Results

The distribution of the rs1815739 *ACTN3* genotype in the sample of women football players is presented in Table 1 ((28.3/52.9/18.8 for RR/RX/GG), which followed HWE. Based on frequency data in the 1000 genome database for the European population (allelic frequency for A/G alleles 56.7/43.7%, respectively), and assuming that this population is in the Hardy–Weinberg equilibrium, the genotype frequencies would be 31.0/50.0/19.0% for RR/RX/XX, respectively. The genotypic frequency in our sample of women football players complies with Hardy–Weinberg, with respect to the 1000 genome database (*p* = 0.871).

Table 2 contains information about the performance tests performed at the beginning of the season. There were no differences in ankle dorsiflexion, sit-and-reach distance, jump height, and sprint time among genotypes. The use of the dominant (RR vs. RX + XX) and recessive models (RR + RX vs. XX) did not produce any statistically significant difference in the performance tests.

During the season, there were recorded a total of 356 non-contact injuries, which represents a mean of 1.86 injuries/player/season. Table 3 contains the number (and frequencies) of players with and without a non-contact injury during the season. The proportion of players with a non-contact injury varied from 75.9 to 83.3% for each genotype without differences among genotypes. The proportions of players with a non-contact muscle injury, ligament injury, and bone injury were also similar among genotypes. The dominant and recessive models did not produce any statistically significant difference in the proportion of players with and without a non-contact injury during the season.

Overall injury incidence was 9.71 injuries per 1000 h of football exposure. Table 4 contains information about injury incidences and the characteristics of the non-contact injuries sustained by the players during the season depending on their *ACTN3* genotype. Injury incidence was between 8.2 ± 5.7 and 10.4 ± 8.6 injuries/1000 h of football exposure, without differences among genotypes. The injury rates during training (from 3.6 ± 3.7 to 4.8 ± 2.1 injuries/1000 h of football training exposure) and during matches (from 47.8 ± 9.5 to 54.1 ± 6.3 injuries/1000 h of football match exposure) were also similar in RR, RX, and XX football players. The distribution of players according to the number of injuries, injury severity, exposure type, recurrence, and mode of onset was similar in RR, RX, and XX football players. The use of the dominant and recessive models did not produce any statistically significant difference in these epidemiological variables.

**Figure 1:**
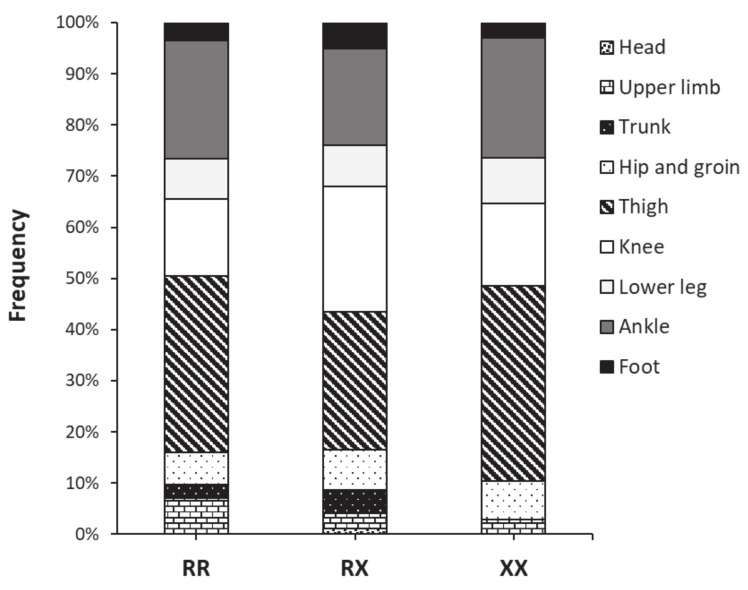
Distribution of non-contact injuries according to their body location in women football players of the first division of Spanish football with different *ACTN3* R577X genotypes.

**Figure 2:**
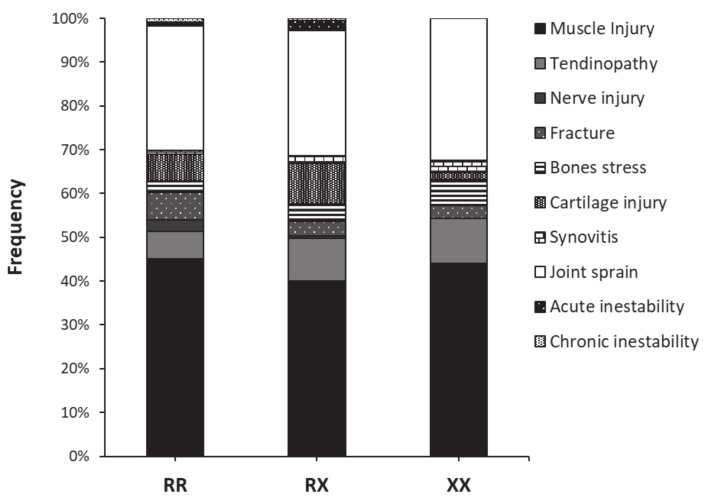
Distribution of non-contact injuries according to their type in women football players of the first division of Spanish football with different *ACTN3* R577X genotypes.

Figure 1 contains information about the body area where the non-contact injuries were located for each genotype. In all three genotypes, the thigh was the most common location for the injury with proportions between 26.9 to 38.2% of the injuries reported within each genotype group. The ankle was the second most common location for RR and XX players, while the knee was the second most common location for RX players. In any case, the distribution of injuries according to the location was not affected by the genotype (*p* = 0.396). Figure 2 depicts the distribution of injuries according to their type in RR, RX, and XX football players. Muscle injury was the most-common injury type in all three genotypes with proportions between 40.0 and 45.1% of the injuries reported within each genotype group. The second most habitual type of injury was joint sprains in all three genotypes, with frequencies between 28.3 and 32.4% of total injuries within each genotype group. The distribution of injuries according to their type was unaltered by the *ACTN3* genotype (*p* = 0.633).

## 4. Discussion

Several previous investigations have found that the *ACTN3* XX genotype, which produces α-actinin-3 deficiency, may be deleterious to becoming a professional football player as the presence of XX players in professional football teams is lower than in control non-athlete populations [16], and they may be more prone to non-contact muscle-type injuries than RR football players [18,19]. However, these findings were obtained in samples of male football players, while the influence of the *ACTN3* R577X polymorphism on women’s football performance has been less investigated [17]. For this reason, the main purpose of this investigation was to determine the influence of the *ACTN3* R577X polymorphism on physical performance variables and injury incidence in a sample of professional women football players. The main outcome of this investigation indicates a negligible influence of *ACTN3* R577X polymorphism on football performance, as the physical performance values and injury rates of RR, RX, and XX women football players were similar. Collectively, the data obtained in this investigation suggest that the *ACTN3* XX genotype may not produce any deleterious phenotype for women football players, contrary to what occurs in men football players.

The assumption that the *ACTN3* XX genotype may produce a deleterious effect on sports performance is supported by several investigations that have reported some potentially negative phenotypes in XX athletes, when compared to RR or R-allele carriers (see these reviews for a more profound analysis [1,2,14]). Briefly, XX athletes may have less muscle strength [30]; lower sprint capacity [12]; higher levels of muscle damage after endurance activities, such as marathons [8] and half Ironman events [31] or eccentric training [32]; and higher probability of muscle injury in team sports [18,19] and endurance sports [33]. Additionally, in samples of active non-athlete individuals, a link has been found between the XX genotype and the presence of ankle injuries [34,35]. There are also studies where the *ACTN3* XX genotype did not influence sports performance [36] and injury rate [37], but they constitute a lower portion than those that found potentially negative phenotypes of the *ACTN3* XX genotype or the X-allele. As it happens in other topics of sports sciences [38], these findings were obtained in samples of male athletes or mixed male/female samples, while the number of investigations on the influence of the *ACTN3* R577X polymorphism in women only athletes is low.

To our knowledge, this is the first experiment that obtains performance and injury data in a sample of women football players of professional teams. In this context, the *ACTN3* XX genotype did not produce any deleterious effect, as the values in the performance tests obtained by XX players were comparable to RR and RX players. Additionally, the injury rates of non-contact injuries or the sub-analysis of muscle, ligament, and bone injuries were similar in XX, RX, and RR players and all within the habitual injury rates found in women football players [39]. Last, the grouping of participants to produce dominant (RR vs. X-allele carriers) and recessive (XX vs. R-allele carriers) models indicated that both R-carriers and X-carriers had similar football-specific performance values and injury rates. These findings contradict the ones found in samples of professional male football players, as an inferior football-specific performance of XX football players has been suggested via underrepresentation of this genotype in professional male football [16] and higher rates of muscle-type injuries in XX players have been found [18,19]. Interestingly, lower sprint and jump capacities of XX football players have been found in professional male players [40], while these capacities measured in women football players with a CMJ and a 30-m sprint test were similar in all three genotypes. Even, in a sample of male football players, it has been shown that no XX players are found in field positions associated with high-speed demands, such as forwards and wingers [41], while the distribution of the RR, RX, and XX genotypes was similar in all the field positions analyzed in the current study in women football players. Although there needs to be confirmatory studies, it seems that the XX genotype may have a less harmful influence on football performance in women than in men.

Despite the reason for the lack of influence of the *ACTN3* XX genotype in women’s football performance is not evident with the data of this study, the different physical, physiological, and tactical characteristics of women’s football—in comparison to men’s football [42]—may have influenced these findings. Existing literature has found that female football players cover less distance and at lower speeds during matches [43] and present lower performance in sprints, jumps, and intermittent endurance than male counterparts [44,45]. As for the tactical side, women’s football teams seem to display a more direct style of play, with fewer passes per possession, and a lower passing tempo than men’s teams [46,47]. These physiological and tactical differences between the characteristics of elite women’s and men’s football may contribute to the insignificance of α-actinin-3 deficiency, due to homozygosity in the *ACTN3* X-allele, found in the present study. Further investigations are warranted to clearly define the *ACTN3* XX genotype as harmless in women football players and other samples of women athletes.

The current study presents some limitations that should be addressed to enhance the application of the results to women’s professional football. Although the study was carried out in a homogeneous sample of female football players participating in the same competition, the sample size is relatively low and not all the teams were subject to identical training, competition, and diet protocols. Additionally, the protocols developed by the staffs of the teams to prevent and to treat injuries were not identical. Therefore, the fact of reaching any definitive conclusions about the lack of association of the *ACTN3* genotype with football performance and injury incidence in professional women players should be made with caution. Future investigations in other samples of professional or elite women athletes should be carried out to replicate the results of this study. Additionally, the current study was focused on only one genetic polymorphism, while sports performance and injury susceptibility may be influenced by other genetic variants not studied in the current experiment. Among others, future investigations should study the potential interaction of different genotypes in target polymorphisms, such as the *Apa*I in the Vitamin D receptor (*VDR*) gene [48], the I/D variation in the angiotensin-converting enzyme (*ACE*) gene [49], and the C-to-T polymorphism in the 3′-untranslated region of the collagen type V α1 chain (*COL5A1*) gene [50], as all of these variants have been found to be associated with some aspect of injury epidemiology in professional football players. Because all these genes may partly contribute to the overall susceptibility of injury in football players, the study of the interaction of the *ACTN3* genotype with other genotypes should include the study of players’ polygenic profile, as in previous studies with other type of athletes [51,52].

## 5. Conclusions

In summary, professional women players with different genotypes of the p.R577X *ACTN3* polymorphism possessed similar values in football-specific performance variables and had very comparable injury incidence rates during training and competition. From a practical perspective, the genotyping of the *ACTN3* gene may not be useful to predict football-specific performance or injury incidence in professional women football players. The influence of this genotype on women’s football seems negligible, at least in comparison to the harmful effect of the *ACTN3* XX genotype found in men football players.

## Figures and Tables

**Table 1:** Age, anthropometric characteristics, field position, level, and football exposure of women football players competing in the first division of Spanish football according to their *ACTN3* R577X genotype.

Variable (Units)	RR	RX	XX	*p* Value
Number (frequency, %)	54 (28.3)	101 (52.9)	36 (18.8)	-
Age (years)	23.8 ± 4.6	23.1 ± 3.9	24.3 ± 4.1	0.304
Height (cm)	167.7 ± 6.1	166.5 ± 6.5	167.0 ± 5.2	0.438
Body mass (kg)	61.7 ± 6.7	59.9 ± 6.3	61.0 ± 5.5	0.220
Body mass index (kg/m^2^)	21.9 ± 2.1	21.6 ± 1.7	21.9 ± 1.6	0.422
Forward (frequency, %)	20 (37.0)	28 (27.7)	9 (25.0)	0.638
Midfielder (frequency, %)	13 (24.1)	29 (28.7)	10 (27.8)	
Defender (frequency, %)	16 (29.6)	31 (30.7)	15 (41.7)	
Goalkeeper (frequency, %)	5 (9.3)	13 (12.9)	2 (5.6)	
International level (frequency, %)	14 (25.9)	23 (22.8)	6 (16.7)	0.586
National level (frequency, %)	40 (74.1)	78 (77.2)	30 (83.3)	
Total exposure time (h)	202 ± 65	212 ± 71	213 ± 78	0.722
Training exposure time (h)	179 ± 64	192 ± 67	189 ± 74	0.614
Match exposure time (h)	23 ± 15	20 ± 14	24 ± 12	0.337

Data are numbers and frequencies (in percentage) or mean ± standard deviation (SD) for each genotype.

**Table 2:** Physical performance variables in women football players competing in the first division of Spanish football according to their *ACTN3* R577X genotype.

Variable (Units)	RR	RX	XX	RR vs. RX vs. XX	Dominant RR vs. RX + XX	Recessive RR + RX vs. XX
Right ankle dorsiflexion (cm)	10.0 ± 2.1	10.6 ± 2.8	9.9 ± 2.2	0.550	0.508	0.522
Left ankle dorsiflexion (cm)	10.3 ± 2.8	10.2 ± 2.0	10.3 ± 2.0	0.992	0.914	0.974
Sit-and-reach distance (cm)	10.9 ± 6.1	7.1 ± 8.1	8.2 ± 7.1	0.361	0.172	0.941
Countermovement jump height (cm)	34.2 ± 5.5	33.8 ± 4.0	33.4 ± 3.5	0.087	0.594	0.599
30 m sprint time (s)	4.80 ± 0.32	4.72 ± 0.77	4.47 ± 0.27	0.210	0.316	0.089

Data are mean ± standard deviation (SD) for each genotype.

**Table 3:** Distribution of women football players of the first division of Spanish football with/without an injury according to their *ACTN3* R577X genotype.

Variable (Units)	RR	RX	XX	RR vs. RX vs. XX	Dominant RR vs. RX + XX	Recessive RR + RX vs. XX
Players with injury (frequency, %)	41 (75.9)	73 (73.3)	30 (83.3)	0.415	0.914	0.219
Players without injury (frequency, %)	13 (24.1)	28 (27.7)	6 (16.7)			
Players with muscle injury (frequency, %)	20 (37.0)	29 (28.7)	15 (41.7)	0.298	0.517	0.250
Players without muscle injury (frequency, %)	34 (63.0)	72 (71.3)	21 (58.3)			
Players with ligament injury (frequency, %)	10 (18.5)	23 (22.8)	10 (27.8)	0.586	0.407	0.401
Players without ligament injury (frequency, %)	44 (81.5)	78 (77.2)	26 (72.2)			
Players with bone injury (frequency, %)	3 (5.6)	8 (7.9)	2 (5.6)	0.811	0.667	0.741
Players without bone injury (frequency, %)	51 (94.4)	93 (92.1)	34 (94.4)			

Data are numbers and frequencies (in percentage) of players with/without injury reported in the preceding season from the total number of players within each genotype.

**Table 4:** Injury incidence, distribution of players according to the number of injuries, and distribution of injuries according to severity, exposure, recurrence and mode of onset in women football players of the first division of Spanish football with different *ACTN3* R577X genotypes.

Variable (Units)	RR	RX	XX	RR vs. RX vs. XX	Dominant RR vs. RX + XX	Recessive RR + RX vs. XX
Incidence						
/1000 h of exposure	10.4 ± 8.6	8.2 ± 5.7	8.9 ± 5.3	0.222	0.112	0.714
/1000 h of training	4.8 ± 2.1	3.6 ± 3.7	3.8 ± 3.5	0.100	0.090	0.401
/1000 h of match	54.1 ± 6.3	51.8 ± 9.4	47.8 ± 9.5	0.209	0.163	0.329
Number of injuries						
No injury (%)	24.1	27.7	16.7	0.074	0.056	0.884
1 injury (%)	37.0	29.7	27.8			
2 injuries (%)	11.1	23.8	25.0			
≥3 injuries (%)	27.8	10.9	16.7			
Return to play						
Severity (days)	39 ± 60	36 ± 65	36 ± 51	0.679	0.543	0.422
Minor (%)	19.5	32.0	29.4	0.127	0.056	0.422
Moderate (%)	45.1	38.9	47.1			
Serious (%)	35.4	29.1	23.5			
Exposure						
Training (%)	40.7	39.4	38.2	0.945	0.772	0.797
Competition (%)	59.3	60.6	61.8			
Recurrence						
New onset (%)	85.8	90.9	92.6	0.261	0.112	0.361
Recurrent (%)	14.2	9.1	7.4			
Mode of onset						
Acute sudden onset (%)	76.1	77.1	79.4	0.795	0.615	0.536
Repetitive gradual (%)	4.4	5.7	7.4			
Repetitive sudden onset (%)	19.5	17.1	13.2			

Data are frequencies (in percentage) from the total of injuries recorded in each genotype and mean ± standard deviation (SD) for each genotype.

## Data Availability

The data presented in this study are available on request from the corresponding author. The data are not publicly available due to legal restrictions.
